# A Case Series of Bosworth Fracture-Dislocations and Review of 129 Reported Cases

**DOI:** 10.5704/MOJ.2211.013

**Published:** 2022-11

**Authors:** C Fang, ZH Tang, CS Yeoh, GM Tan

**Affiliations:** Department of Orthopaedic Surgery, Khoo Teck Puat Hospital, Singapore

**Keywords:** bosworth, fracture-dislocation, irreducible, ankle

## Abstract

**Introduction:**

Bosworth fracture dislocations of the ankle are rare injuries of the ankle caused by extreme external rotation of the supinated foot where the proximal fibula fracture fragment is posteriorly dislocated and entrapped behind the posterior-lateral ridge of the tibia. This case series aims to document three such cases treated in our institution over a nine year period. We also provide a review of 129 cases in the existing literature.

**Materials and methods:**

Medical records and relevant radiographs for each patient were analysed and collected from the time of presentation till the point of latest follow-up. During each clinic visit, all physical exam findings as well as all complications were recorded. The American Foot and Ankle Society (AOFAS) Hindfoot score was also tabulated for each patient at the point of latest review.

**Results:**

Closed reduction was unsuccessful in all three patients, and all required open reduction. One patient had an uncomplicated recovery whilst the remaining two suffered significant soft tissue complications. One patient suffered severe soft tissue swelling preventing primary closure at the time of surgery, whilst another suffered post-operative wound dehiscence and infection. Eventually all fractures healed, and all three patients obtained satisfactory AOFAS scores.

**Conclusion:**

The diagnosis of Bosworth fracture dislocations of the ankle is often delayed or missed, due to its rare occurrence. Closed reduction is often unsuccessful, and early open reduction is required to avoid poor clinical outcomes due to severe soft tissue damage or even compartment syndrome.

## Introduction

The Bosworth fracture dislocation refers to a rare pattern of fibula fractures where the proximal fibula fragment is posteriorly dislocated and entrapped behind the posterior-lateral ridge of the tibia. In a retrospective review of 3405 patients, one paper reported its prevalence to be approximately 1.6%^1^.

The key to successful treatment of Bosworth fracture dislocations involves early recognition and diagnosis of the injury, followed by prompt open reduction^2,3^ as they are often irreducible using closed reduction techniques, and delayed recognition and reduction results in higher complication rates and poor outcomes.

Our study’s primary aim is to contribute and report the presentation, treatment, and outcomes of three such cases, of which one is previously reported^2^, but included with the purpose of reporting long-term outcomes.

The secondary aim of our study was to review and summarise the existing literature to achieve the following key objectives. Firstly, we aimed to collate reported cases of Bosworth fracture-dislocations to better understand the nature of the bony and soft tissue injuries associated with the injury, as well as the incidence of associated complications. Secondly, we aimed to provide a summary of any existing diagnostic and treatment techniques previously reported to give the reader an overview of the topic.

## Materials and Methods

This study is a retrospective case series. All three patients in our series were treated in our institution from the period of 2011 to 2019 and were operated on by the senior authors of this study where the diagnosis of Bosworth type fracture dislocations was confirmed intra-operatively.

For each patient, the intra-operative surgical technique, as well as subsequent treatment including the frequency of follow-up clinic visits were not standardised and were left to the discretion of the attending primary surgeon. All three patients had standardised formal post-operative physiotherapy and occupational therapy sessions as per our institutions protocol.

For the study, we retrospectively reviewed all clinical notes, radiographs, and intra-operative notes for each of the three patients. During each subsequent clinic visit, all physical exam findings including ankle range of motion as well as all complications were specifically recorded. The American Foot and Ankle Society (AOFAS) Hindfoot score was also tabulated for each patient at the point of latest review.

Our literature review was performed using the Pubmed, EMBASE and Google Scholar databases using the following search terms: Bosworth fracture, Bosworth injury, Bosworth fracture-dislocation. All full-length articles published in English pertaining to Bosworth fracture-dislocations of the ankle were read in entirety. Studies reporting variants of the Bosworth fracture-dislocation such as Reverse-Bosworth lesions and Bosworth-Pilon injuries were excluded. All references were also checked to ensure no cases were duplicated. Two studies we identified were not included despite meeting the inclusion criteria as we were unable to assess the full article through our institutions research databases.

## Results

We treated a total of 3 patients with Bosworth fracture - dislocations of the ankle, consisting of 2 males and 1 female with a mean age of 35 years old (32–37 years old. All were young, healthy patients with no significant past medical history.

Of the 3 patients, 2 (Patient 1 and Patient 3) were football injuries, whilst the final patient (Patient 2), suffered a twisting injury to her ankle while tying her shoelaces. The injury mechanism was supination-external rotation in all three patients.

All three injuries were closed injuries, and all patients were neurovascularly intact on arrival. In all three patients, the diagnosis of a Bosworth fracture-dislocation was made only after an initial attempt at closed reduction was unsuccessful. A further unsuccessful attempt at closed reduction was attempted in two out of three patients (Patient 1 and Patient

2) prior to surgery, whilst the last patient was immediately counselled for emergent open reduction and fixation. None of our patients had pre-operative computer tomography scans, and only one patient (Patient 1), had a scan postoperatively to confirm syndesmotic reduction.

Two out of three patients (Patient 2 and Patient 3) were operated within 8 hours from the timing of surgery, whilst the last patient (Patient 1) underwent surgery 26 hours post injury. Table I summarises the intra-operative findings for all three patients. Intra-operatively, the proximal fibula fracture fragment was confirmed to be incarcerated posterior to the posterior-lateral ridge of the tibia requiring open reduction for all three patients. For reduction, either a curved osteotome or a Hoffman retractor was utilised to lever the entrapped proximal fibula free from the postero-lateral ridge of the tibia. All patients sustained Weber type B fibula fractures with the fracture line running from a posterosuperior to anterior-inferior direction.

**Table I:** Intra-operative findings

Patient	Weber	Fibula fracture	Medial Malleolus	Posterior Malleolus	Chaput Fragment	Syndesmotic ligaments	Deltoid ligament
1 36 y/o Male	B	Oblique fracture pattern Posterior-superior proximally to Anterior-inferior	Intact	Nil	Nil	Complete disruption of syndesmotic complex	Intact
2 37 y/o Female	B	Short oblique fracture Posterior-superior proximally to Anterior-inferior distally	Intact	Yes <25%	Nil	Complete disruption of anterior syndesmotic ligaments	Disrupted
3 32 y/o Male	B	Oblique fracture Posterior-superior proximally to Anterior-inferior distally	Intact	Nil	Nil	Complete disruption of syndesmotic complex	Intact

Post-anatomical reduction of the fibula, the fibula fractures were fixed either using a lag screw and neutralisation plate construct, or locking one-third tubular plates. For all three fractures, a single syndesmotic screw was utilised for syndesmotic stabilisation after a positive cottons test post fibula-fixation. In one patient (Patient 2), the anterior syndesmotic ligaments were repaired primarily with Vicryl 1-0 sutures.

**Fig. 1.**
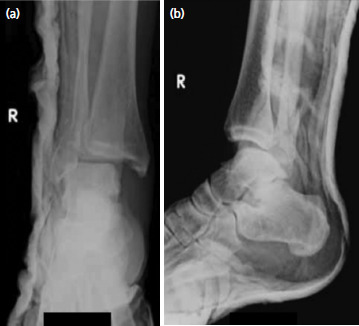
Patient 1 – (a) Antero-posterior and (b) lateral radiographs on arrival showing a short oblique lateral malleolar fracture and postero-lateral subluxation of the talus with entrapment of the proximal fibula behind the tibia.

**Fig. 2.**
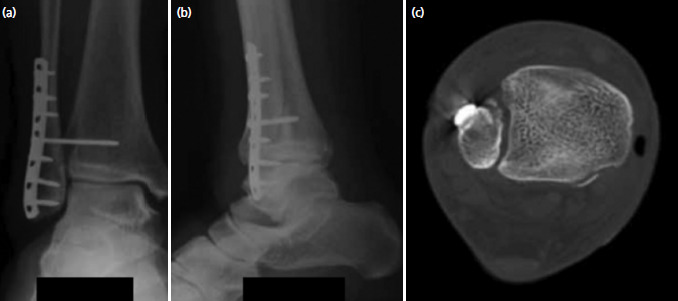
Patient 1 – Post-operative (a) antero-posterior, (b) lateral and (c) axial computer tomography of the right ankle joint showing adequate reduction.

**Fig. 3.**
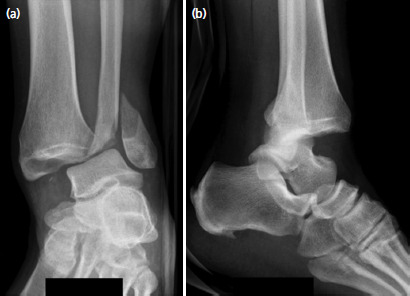
Patient 2 – Pre-reduction (a) antero-posterior and (b) lateral radiographs of the ankle illustrating a Weber type B lateral malleolus fracture and dislocation of the ankle joint.

**Fig. 4.**
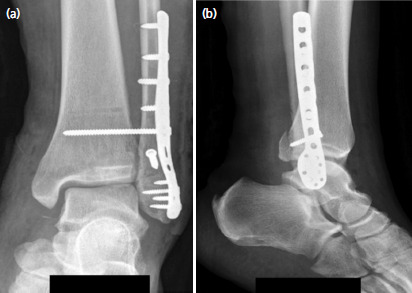
Patient 2 – Post-operative (a) antero-posterior and (b) lateral radiographs of the ankle at six weeks.

**Fig. 5.**
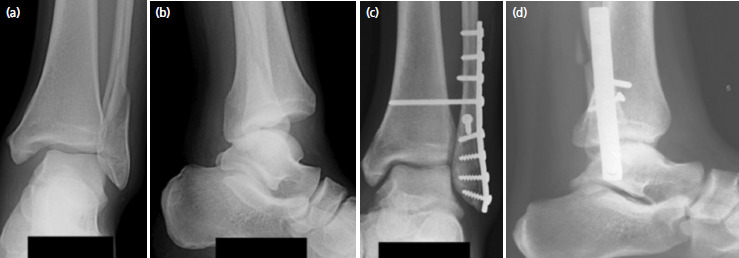
Patient 3 – (a, b) Pre-operative and (c, d) post-operative. (a) Antero-posterior and (b) lateral films.

Post-operatively, all patients were kept non-weight bearing in a short walker boot for six weeks, before progressive weight bearing was commenced. Fig. 1 to 5 illustrate the preoperative and post-operative radiographs for each patient.

Two out of three patients suffered soft tissue complications. The first patient (Patient 1), had significant soft tissue swelling preventing primary closure at the time of surgery. In lieu of this, the periosteum was closed over the fibula plate and the wound was temporarily tagged with prolene sutures and covered with a negative pressure dressing. Successful coverage was obtained 10 days later with a split thickness skin graft, and the patient subsequently had an uncomplicated recovery. The second patient (Patient 2) was noted to have had post-operative cellulitis and wound dehiscence two weeks post-operatively. She was successfully treated conservatively with oral antibiotics and subsequently recovered well.

Despite two out of three patients suffering soft tissue complications, all three fractures united and all three patients recovered well with excellent functional outcome scores (AOFAS Hindfoot scores) at the point of last follow-up. At the point of latest follow-up, the mean AOFAS score for all three patients was 99.3. All three patients reported no limitation in function inclusive of recreational sporting activities (Table II). There were no cases of compartment syndrome, non-union, malunion or secondary osteoarthritis in our series of patients.

**Table II:** Patient demographics and outcomes.

Patient	Weber Type/ Mechanism	Closed reduction	No. of attempts	Injury to surgery (hours)	Complications	AOFAS Hindfoot Score	Outcomes (At least review)
1	B / SER	Unsuccessful	2	26	Severe post-operative swelling not amenable to primary closure	100 points (9 years post-op)	No chronic pain or instability Back to playing football
2	B / SER	Unsuccessful	2	6	Post-operative cellulitis and wound dehiscence	100 points (9 months post-op)	No chronic pain or instability Went back to work
3	B / SER	Unsuccessful	1	8	Nil	98 points (5.2 years post-op)	No chronic pain Infrequent ankle instability Back to running

## Discussion

The classical Bosworth fracture-dislocation is a fibula dislocation associated with a fibula fracture where the proximal fibula fracture fragment is incarcerated behind the postero-lateral ridge of the tibia, held rigid by the tension of the interosseous membrane. This is usually associated with either a rupture of the deltoid ligament or a fracture of the medial malleolus^3^.

Several variants of the injury exist. The first is a dislocation of the distal fibula associated with epiphyseolysis of the distal tibia, which occurs in children with open physeal plates. Other variants are the Bosworth lesion^4^ which refers to a distal fibula dislocation without fracture (usually in young adults), and the “Reverse Bosworth Lesion”^5^ where in Weber type C fractures the distal fibula fragment instead of the proximal fibula fragment gets incarcerated behind the posterior tubercle of the tibia. Lastly, “Bosworth-Like” lesions referring to fibula dislocations associated with a tibial pilon fracture have also been described^6-8^.

Perry *et al*^9^ anatomical study published in 1983 described seven stages of injury beginning with rupture of the anterior tibiofibular ligament followed by sequentially, rupture of the posterior tibiofibular ligament, antero-medial capsule and finally inter-osseous membrane. Following this, the intact lateral collateral ligament then dislocates the fibula posteriorly causing entrapment behind the postero-lateral ridge of the tibia, and fracture of the fibula then occurs with persistence of a supination / external rotation stress as the talus rotates. The final step is then either fracture of the medial malleolus or rupture of the deltoid ligament.

To better understand the nature of the injury, we reviewed a total of 31 publications^1,4,9,10-37^ collectively describing 129 patients with Bosworth fracture-dislocations of the ankle.

Amongst these patients, a supination-external rotation injury mechanism was the most common (75.7%), whilst the next most commonly mechanism was due to falls from height (8.7%). High energy sprains (6.8%), low energy sprains (5.8%) and pronation external rotation injuries (2.9%) made up the remaining reported mechanisms. Twenty-six out of 129 patients had no clear mechanism of injury documented (Table III).

**Table III:** Mechanism of injury

Author / Year	Cases	Mechanism of injury	Comments
Bartonick *et al*, 2022^10^	1	SER:0, PER:0, FH:0, HS:1, LS:0, NA:0	Sprained whilst running
Han *et al*, 2021^11^	1	SER:0, PER:0, FH:0, HS:1, LS:0, NA:0	Slipped going down mountain
He *et al*, 2020^12^	1	SER:0, PER:0, FH:0, HS:0, LS:0, NA:1	Road traffic accident
Wang *et al*, 2020^13^	1	SER:0, PER:0, FH:0, HS:0, LS:0, NA:1	-
Martin-Somoza *et al*, 2020^14^	1	SER:0, PER:0, FH:1, HS:0, LS:0, NA:0	Fall down stairs
Fan *et al*, 202015	1	SER:1, PER:0, FH:0, HS:0, LS:0, NA:0	Skateboarding injury
Kostlivy *et al*, 2020^4^	13	SER:0, PER:0, FH:0, HS:0, LS:0, NA:13	-
Ren *et al*, 2019^16^	2	SER:1, PER:1, FH:0, HS:0, LS:0, NA:0	1 Fall from bike, 1 Rugby injury
Won *et al*, 2019^1^	51	SER:51, PER:0, FH:0, HS:0, LS:0, NA:0	Retrospective review of 3405 SER type #
Cho *et al*, 2019^17^	15	SER:13, PER:2, FH:0, HS:0, LS:0, NA:0	-
Foldager *et al*, 2018^18^	2	SER:2, PER:0, FH:0, HS:0, LS:0, NA:0	1 Football, 1 Fall from standing height
Williams *et al*, 2018^19^	1	SER:0, PER:0, FH:1, HS:0, LS:0, NA:0	Jumped into lake
Saraiva *et al*, 2016^20^	1	SER:1, PER:0, FH:0, HS:0, LS:0, NA:0	-
Downey *et al*, 201621	5	SER:4, PER:0, FH:0, HS:0, LS:0, NA:1	-
Hancock, 2015^22^	1	SER:0, PER:0, FH:1, HS:0, LS:0, NA:0	Fall down stairs
Silverio *et al*, 2014^23^	1	SER:1, PER:0, FH:0, HS:0, LS:0, NA:0	Football injury
Yang *et al*, 2014^24^	4	SER:0, PER:0, FH:0, HS:2, LS:2, NA:0	2 Sporting injury, 2 Fall from standing height
Delasotta *et al*, 2013^25^	1	SER:0, PER:0, FH:0, HS:1, LS:0, NA:0	Football injury
Ellanti *et al*, 2013^26^	1	SER:0, PER:0, FH:0, HS:0, LS:0, NA:1	-
Schepers *et al*, 2012^27^	1	SER:0, PER:0, FH:0, HS:0, LS:1, NA:0	Fall from stairs
Wright *et al*, 2012^28^	1	SER:1, PER:0, FH:0, HS:0, LS:0, NA:0	Football injury
Khan *et al*, 2008^29^	1	SER:1, PER:0, FH:0, HS:0, LS:0, NA:0	Dancing with heels
Lui *et al*, 2008^30^	4	SER:0, PER:0, FH:2, HS:1, LS:1, NA:0	2 Fall from stairs, 1 Fall from standing height, 1 Ice-Skating
Bartonicek *et al*, 2007^31^	6	SER:0, PER:0, FH:3, HS:1, LS:2, NA:0	1 Fall from stairs, 1 Fall from bike, 1 Fell into ditch, 1 Football injury, 2 Fall from standing height
Chung *et al*, 2004^32^	1	SER:0, PER:0, FH:0, HS:0, LS:0, NA:1	-
Beekman *et al*, 2003^33^	1	SER:1, PER:0, FH:0, HS:0, LS:0, NA:0	Football injury
Jehlicka *et al*, 2001^34^	1	SER:0, PER:0, FH:0, HS:0, LS:0, NA:1	-
Szalay *et al*, 2001^35^	1	SER:0, PER:0, FH:0, HS:0, LS:0, NA:1	-
Molinari *et al*, 1990^36^	1	SER:1, PER:0, FH:0, HS:0, LS:0, NA:0	Fell whilst hang-gliding
Perry *et al*, 198^39^	2	SER:0, PER:0, FH:1, HS:0, LS:0, NA:1	1 Intoxicated patient, 1 Fell from 5 feet
Bosworth, 1947^37^	5	SER:0, PER:0, FH:0, HS:0, LS:0, NA:5	-
**Total**	**129**	**SER:78, PER:3, FH:9, HS:7, LS:6, NA:26**	

Abbreviations - SER: Supination External rotation, PER: Pronation External Rotation, FH: Fall from height*, HS: High energy sprain, LS: Low energy sprain, NA: Not Clearly Documented

*fall from height defined as any height above waist level

The vast majority of fibula fractures were Weber type B fractures (83.5%). The remaining patients had Weber type C fractures (12.6%) or had no associated fibula fracture (3.2%). Only 1 patient sustained a Weber type A injury^1^.

Eliminating articles where the presence or absence of the relevant fracture pattern was not clearly documented, a posterior malleolus fracture was noted in 58.4% (69/118) of patients, whilst medial malleolus fractures was noted in 52.4% (55/105). Chaput fragments were documented in 16.2% of fractures (17/105) and only 2 patients had Wagstaffe fragments.

Of the patients reviewed, only a handful of patients had computer tomography scans pre-operatively, and some authors have postulated^4^ that the incidence of posterior malleolar fractures in Bosworth fracture-dislocations could be under-reported as a result.

The incidence of deltoid rupture was 37.5% (27/72), although a large number of studies (21/31) did not explicitly comment on the integrity of the deltoid ligament (Table IV). A high index of clinical suspicion and awareness of the condition is needed as these injuries are rare and often missed. Clinically, an irreducible ankle associated with a externally rotated foot should immediately raise the possibility of such an injury.

**Table IV:** Fracture configuration

Author / Year	Cases	Weber	PM	MM	Chaput Fragment	Wagstaffe Fragment	Deltoid rupture
Bartonick *et al*, 2022^10^	1	A:0, B:1, C:0, No #:0	1	-	-	-	NA
Han *et al*, 2021^11^	1	A:0, B:1, C:0, No #:0	-	-	-	-	-
He *et al*, 2020^12^	1	A:0, B:0, C:1, No #:0	1	-	-	-	1
Wang *et al*, 2020^13^	1	A:0, B:1, C:0, No #:0	1	-	-	-	NA
Martin-Somoza *et al*, 2020^14^	1	A:0, B:1, C:0, No #:0	1	1	-	-	-
Fan *et al*, 2020^15^	1	A:0, B:1, C:0, No #:0	-	1	-	-	NA
Kostlivy *et al*, 2020^4^	13	A:0, B:8, C:5, No #:0	13	NA	NA	NA	NA
Ren *et al*, 2019^16^	2	A:0, B:1, C:0, No #:1	2	2	-	-	NA
Won *et al*, 2019^1^	51	A:1, B:48, C:02, No #:0	29	28	9	2	22
Cho *et al*, 2019^17^	15	A:0, B:13, C:2, No #:0	9	13	3	-	NA
Foldager *et al*, 2018^18^	2	A:0, B:2, C:0, No #:0	1	1	-	-	NA
Williams *et al*, 2018^19^	1	A:0, B:0, C:0, No #:1	-	-	-	-	NA
Saraiva *et al*, 2016^20^	1	A:0, B:1, C:0, No #:0	1	1	-	-	NA
Downey *et al*, 2016^21^	5	A:0, B:4, C:0, No #:1	NA	NA	NA	NA	NA
Hancock, 2015^22^	1	A:0, B:1, C:0, No #:0	1	-	-	-	NA
Silverio *et al*, 2014^23^	1	A:0, B:1, C:0, No #:0	-	-	-	-	NA
Yang *et al*, 2014^24^	4	A:0, B:4, C:0, No #:0	-	3	-	-	1
Delasotta *et al*, 2013^25^	1	A:0, B:1, C:0, No #:0	1	-	1	-	NA
Ellanti *et al*, 2013^26^	1	A:0, B:0, C:1, No #:0	-	-	-	-	NA
Schepers *et al*, 2012^27^	1	A:0, B:1, C:0, No #:0	-	-	-	-	-
Wright *et al*, 2012^28^	1	A:0, B:1, C:0, No #:0	1	-	-	-	NA
Khan *et al*, 2008^29^	1	A:0, B:1, C:0, No #:0	1	-	-	-	-
Lui *et al*, 2008^30^	4	A:0, B:4, C:0, No #:0	-	1	-	-	-
Bartonicek *et al*, 2007^31^	6	A:0, B:3, C:3, No #:0	6	2	3	-	3
Chung *et al*, 2004^32^	1	A:0, B:1, C:0, No #:0	NA	NA	NA	NA	NA
Beekman *et al*, 2003^33^	1	A:0, B:1, C:0, No #:0	-	-	-	-	NA
Jehlicka *et al*, 2001^34^	1	A:0, B:0, C:1, No #:0	-	1	-	-	NA
Szalay *et al*, 2001^35^	1	A:0, B:1, C:0, No #:0	NA	NA	NA	NA	NA
Molinari *et al*, 1990^36^	1	A:0, B:0, C:1, No #:0	NA	NA	NA	NA	NA
Perry *et al*, 1983^9^	2	A:0, B:1, C:0, No #:1	-	-	1	-	-
Bosworth, 1947^37^	3	A:0, B:3, C:0, No #:0	-	1	-	-	NA
**Total**	**127**	**A:1, B:106, C:16, No #:4**	**69**	**55**	**17**	**2**	**27**

Abbreviations - PM: Posterior malleolus, MM: Medial malleolus, NA: Not Clearly Documented, - : None involved

Although several radiographic signs have been reported, these can be limited by poor radiographic projections especially if the patient is in significant discomfort. In lieu of this, some clinicians^1,28^ advocate for the routine use of computer tomography scans if expediently available and does not delay open reduction.

Khan and Borton^29^ described the “Axilla Sign” on mortise views of the ankle, which refers to a visible radiodensity at the axilla of the medial tibial plafond due to persistent internal rotation of the tibia (caused by the incarcerated fibula fragment). Although the axilla sign performed well in his original study, it was apparent in only one out three patients in our series.

Another study by Yang *et al*^24^, described using external oblique radiographs to assess the degree of posterior fibula displacement relative to the length of the talus. In their series of four cases, a line drawn parallel to the shaft proximal fibula fracture transacting near the midpoint of the length of the talus (indicating posterior fibula displacement) was diagnostic. Early recognition and open reduction is the standard of care in such injuries mainly due to abysmal success rates with closed reduction.

Fan *et al*^15^, reported success with a two-man reduction technique. With the patients knee flexed, and the ankle dorsiflexed with traction, successful reduction was obtained with one provider giving an anterior force to the talus with supination and internal rotation of the foot, and another applying a lateral and anterior force to the postero-medial surface of the proximal fibula shaft with a stabilising medial counter-force over the tibia.

Prior to that, Mayer and Evarts^38^ also described their technique which involved traction and medial rotation of the foot together with a laterally directed force over the proximal fibula shaft with the patient under general anaesthesia. Bartonicek *et al*^31^ reported success with simple pulling and gradual internal rotation of the foot. All aforementioned cases of successful closed reduction was associated with an audible “snap” on reduction.

Although these are useful techniques worthwhile attempting especially in centres where surgical expertise may not be readily available, we emphasise that multiple attempts should be avoided to reduce the rates of soft tissue complications and compartment syndrome.

Most cases of open reduction utilised a myriad of tools such as a hemostat or Hoffmans retractor to lever the fibula away from the tibia. The postero-lateral approach to the fibula is recommended in such injuries as the conventional lateral approach makes access to the dislocated fibula challenging^26^, and also allows for better access to the posterior malleolus.

If reduction cannot be obtained despite adequate visualisation, an additional incision over the antero-medial ankle can be considered to assess for soft tissue interposition of the medial ankle structures. In one study^27^, a large slip of the anterior capsule was noted to have ruptured and was lodged in between the tibia and talus. Additionally, the ankle should also be examined for bony fragments from the medial malleolus, posterior malleolus or ankle joint. If an associated Volkmann fracture is present, rarely the proximal fibula shaft fragment can incarcerate between the posterior tibial lip fragments, requiring considerable effort to achieve reduction^28,39^.

Once reduction is obtained, the resulting fracture can be fixed in the typical fashion. If a medial malleolar fracture is present or if there is suspicion of deltoid ligament injury, a medial incision and approach can be utilised.

To date, there are no existing papers which advocate for routine deltoid exploration or adjunct repair of the deltoid ligament. In all three of our patients, syndesmotic fixation alone was sufficient to stabilise the ankle mortise.

As an adjunct, ankle arthroscopy can be considered to assess the syndesmosis and subsequent adequacy of reduction (if needed) and also evaluate for the presence of associated intra-articular pathology^30^.

The most common complications associated with Bosworth fracture-dislocations are soft tissue complications such as infection and severe lower limb swelling which occurred in two out of three out of patients. In our series, the delayed time to surgery and fracture reduction (26 hours in Patient 1) resulted an unopposable surgical wound, which required temporary coverage with a negative pressure dressing and eventually split thickness skin grafting^2^.

Severe complications include osteoarthritis of the ankle and compartment syndrome. In our review, the incidence of osteoarthritis was 13.2% (16/121) although this is likely under-reported due to the short length of follow-up for most studies. In one study with long term follow-up^30^, the incidence of osteoarthritis was 75% (3/4 patients).

Compartment syndrome occurred in 8 patients^1,10,16,17,32,33,35^ (6.2%) which is significant due to the relative rarity of compartment syndrome in typical ankle fractures. In two cases^10,33^, compartment syndrome occurred pre-operatively due to failure of reduction that was undiagnosed. In 5 patients^1,16,32,35^ compartment syndrome occurred postoperatively, of which all except 1 (diagnosed 8 days postoperatively)^32^ was diagnosed acutely between 8 hours and 24 hours post-operatively. The timing of compartment syndrome was not documented in one patient^17^. Urgent fasciotomy was performed in all cases of acute compartment syndrome. Amongst all 8 patients, only 1 patient^16^ obtained normal function at 18 months, with the rest reporting complications such as residual weakness, stiffness and contracture. In lieu of this, it is imperative that the attending clinician ensure that satisfactory reduction is obtained, and also be vigilant to the fact that compartment syndrome can occur post-operatively despite adequate reduction especially if the patient underwent multiple failed attempts at closed reduction, had severe deformity on presentation, or had significant delay to surgery (>24 hours)^1^.

## Conclusion

A Bosworth fracture-dislocation of the ankle must be considered in the event of an irreducible ankle fracture. Successful management of these injuries relies on making an early diagnosis followed by prompt open reduction should closed reduction fail. Repeated attempts at closed reduction are likely to fail and should be avoided. Although computer tomography scans can be obtained pre-operatively to aid surgical planning, it is not strictly necessary and should only be performed if it does not result in a significant delay to surgery. If early diagnosis and reduction is achieved, in the absence of compartment syndrome satisfactory clinical outcomes are expected.
